# Clinical Application of Chinese Herbal Injection for Cancer Care: Evidence-Mapping of the Systematic Reviews, Meta-analyses, and Randomized Controlled Trials

**DOI:** 10.3389/fphar.2021.666368

**Published:** 2021-05-07

**Authors:** Ming Yang, Si-jia Zhu, Chen Shen, Rui Zhai, Duo-duo Li, Min Fang, Jing-nan Xu, Ye-na Gan, Lu Yang, Zhi-ying Ren, Ruo-xiang Zheng, Nicola Robinson, Jian-ping Liu

**Affiliations:** ^1^ Centre for Evidence-based Chinese Medicine, Beijing University of Chinese Medicine, Beijing, China; ^2^ Dongzhimen Hospital, Beijing University of Chinese Medicine, Beijing, China; ^3^ Third Affiliated Hospital, Beijing University of Chinese Medicine, Beijing, China; ^4^ Institute of Acupuncture and Moxibustion in Cancer Care, Beijing University of Chinese Medicine, Beijing, China; ^5^ Beijing University of Chinese Medicine, Beijing, China; ^6^ China-Japan Friendship Hospital, Beijing, China; ^7^ Institute of Health and Social Care, London South Bank University, London, United Kingdom; ^8^ Institute of Integrated Traditional Chinese Medicine and Western Medicine, Guangzhou Medical University, Guangzhou, China

**Keywords:** chinese herbal injection, cancer care, systematic review, randomized controlled trial, clinical application, herbal medicine, evidence mapping, evidence-based decision-making

## Abstract

**Background and objective:** Cancer is a life-threatening disease worldwide and current standard therapy cannot fulfill all clinical needs. Chinese herbal injections have been widely used for cancer in Chinese and Western hospitals in China. This study aimed to apply evidence mapping in order to provide an overview of the clinical application of Chinese herbal injections in cancer care based on randomized controlled trials, systematic reviews, and meta-analyses.

**Methods and results:** Seven databases were systematically searched for eligible randomized controlled trials, systematic reviews, and meta-analyses for ten Chinese herbal injections used in cancer treatment and covered in the Chinese national essential health insurance program. Excel 2016 and RStudio were used to integrate and process the data.

In total 366 randomized controlled trials and 48 systematic reviews and meta-analyses were included in the evidence mapping of herbal medicines including; Compound Kushen, Shenqi Fuzheng, Aidi, Kangai, Kanglaite, Xiaoaiping, Cinobufacin, *Brucea javanica* oil emulsion, *Polyporus* polysaccharide injection, and *Astragalus* polysaccharide for injection. Health insurance restricts the scope of clinical application for these herbal injections. The numbers of studies published increased, especially around 2013–2015. The most studied cancer types were lung cancer (118, 32.2%), colorectal cancer (39, 10.7%), and gastric cancer (39, 10.7%), and the most used injections were Compound Kushen (78, 21.3%), Shenqi Fuzheng (76, 20.8%), and Aidi (63, 17.2%). The most consistently reported benefits were observed for Compound Kushen, Shenqi Fuzheng, Aidi, and Kangai for tumor response, quality of life, myelosuppression, and enhancing immunity.

**Conclusion:** The current evidence mapping provides an overview of the outcomes and effects of Chinese herbal injections used in cancer care, and offers information on their clinical application which warrants further evidence-based research in order to inform clinical and policy decision-making.

## Introduction

Cancer is one of the most common diseases worldwide. The global cancer burden was estimated to have risen to 19.3 million new cases with 10.0 million deaths in 2020 (The International Agency for Research on Cancer (IARC), 2020). Currently, western anti-cancer medications are the dominant treatment approach, but they cannot meet all clinical needs in cancer care. As statistics show, complementary and alternative therapies fulfill a great demand in cancer treatment (Horneber et al., 2012), and the evidence-based clinical practice of these therapies for cancer is growing and being recommended (Greenlee et al., 2017).

In the field of traditional Chinese medicine (TCM), herbal injections were developed in the 1940s and became an important component of Chinese patent herbal drugs (Li et al., 2018), and widely used in both Chinese medicine and Western medicine hospitals in China. By 2015, the Chinese herbal injection industry had reached 87.706 billion RMB (Rui Zheng et al., 2018). However, with the strengthening of the reform of public hospitals, the government has introduced a series of policies on the use of adjuvant drugs and are dynamically adjusting the healthcare insurance catalogue (National Health Commission, 2018; General Office of the National Health Commission, Office of the National Administration of Traditional Chinese Medicine, 2019). Given the advent of strict regulations, the market share of Chinese herbal injection declined rapidly. Evidence-based research is urgently needed to support the rational and valuable use of Chinese herbal injections in clinical practice.

An increasing number of randomized clinical trials (RCTs), systematic reviews (SRs), and meta-analyses on Chinese herbal injections for cancer care have been published in recent decades. However, a large quantity of the clinical studies have used inappropriate evidence synthesis, and a wide range of conclusions on the different injections has made consensus on the clinical utility of injections ambiguous. Evidence mapping is a systematic search of a broad field which enables a comprehensive analysis of the topic and can identify priority areas for future research (Miake-Lye et al., 2016).

This study aimed to map the existing evidence from RCTs, systematic reviews, and meta-analyses for ten Chinese herbal injections which are categorized for use in cancer care in the national basic medical insurance program in China, and to provide precise indications for the clinical use of these injections.

## Methods

### Inclusion Criteria

All RCTs, SRs, and meta-analyses (except network meta-analyses) on Chinese herbal injections for cancer care were included. The Chinese herbal injections included were as follows: Compound Kushen, Shenqi Fuzheng, Aidi, Kangai, Kanglaite, Xiaoaiping, Cinobufacin, *Brucea javanica* oil emulsion, *Polyporus* polysaccharide injection and *Astragalus* polysaccharide for injection. The included RCTs had to state the inclusion criteria of the participants and appropriate randomization methods as this would lead to a low risk of bias in the random sequence generation assessment (Higgins et al., 2011). Based on part of the critical domains in AMSTAR2 (A MeaSurement Tool to Assess systematic Reviews) (Shea et al., 2017), the included SRs and meta-analyses should have conducted an adequate search of at least two databases, assessed the risk of bias from individual studies being included in the reviews, used appropriate methods of meta-analysis, considered the risk of bias when interpreting the results of the reviews, and if possible assess the presence and likely impact of publication bias.

Participants had to be diagnosed as having a malignant tumor according to their pathology and there were no restrictions on age, gender, types of cancer, stages, complications, or previous treatment. Types of interventions were defined as those with at least one arm with one of the herbal injections in the national essential health insurance program as either anti-cancer or adjuvant treatment for cancer (ZC01 and ZC02 category, 2019 version) including those currently in the access negotiation list during the agreement period (National Health Insurance Agency, 2019). There were no limitations on dosage, duration, and any combined therapy should be standard therapy following the NCCN (National Comprehensive Cancer Network) guideline without other TCM interventions. The control group could be standard anti-cancer treatment, placebo, or no treatment without other TCM therapies. Primary outcomes included survival, tumor response, or quality of life; secondary outcomes included cancer-related symptoms, side-effects of chemo/radiotherapy or targeting therapy, and immune function. No limitations were imposed on study setting, language, or publication type. The articles with only one author except for dissertation and conference abstracts without details were excluded.

### Search Strategy

We searched PubMed, EMBASE, Web of Science, and four major Chinese electronic databases, China National Knowledge Infrastructure (CNKI), Chinese Scientific Journals Database (VIP), Chinese BioMedical Literature Database (SinoMed), and Wanfang Database, from their inception to Dec 2nd, 2020. Ten herbal injections - Compound Kushen, Shenqi Fuzheng, Aidi, Kangai, Kanglaite, Xiaoaiping, Cinobufacin, *Brucea javanica* oil emulsion, *Polyporus* polysaccharide injection and *Astragalus* polysaccharide for injection - were searched independently. Taking Aidi injection as an example, the following search strategy was applied in PubMed:

#1 (Neoplasm) OR (Carcinoma) [MeSH Terms]
#2 (Cancer*) OR (Tumor*) OR (Tumour*) OR (Malignant*) [Title/Abstract]
#3 #1 OR #2
#4 (Aidi injection) [Title/Abstract]
#5 (random*) [Text Word]
#6 (meta) OR (systematic review) OR (review) [Title/Abstract]
#7 #5 OR #6
#8 #3 AND #4 AND #7
#9 (mice) or (mouse) or (rat) or (rabbit) [Title]
#10 #8 NOT #9

### Study Selection and Data Extraction

The authors (SJZ and CS) screened the titles and abstracts of all retrieved references after removing duplicates, and the full-text was retrieved for further screening. MY checked all the eligible studies and any disagreements were solved by discussion with SJZ and CS. The data extraction was conducted by RZ, MF, YNG, LY, ZYR, CS, SJZ, MY, and RXZ and validated by SJZ and MY. The extracted information recorded was authors, publication year, title, funding, randomization methods, single/multiple centers, blinding, sample size, cancer type and stage, the TCM syndrome classification, the controls, the administration, dosage, duration of herbal injection, and the outcomes.

### Data Synthesis

The basic information on the ten injections was summarized according to the drug indications and the national essential health insurance program list (2019 version) (National Health Insurance Agency, 2019). The quantitative description and figures were conducted with Microsoft Excel 2016 and RStudio statistical software (RStudio; Boston, Massachusetts, United States).

## Results

### The Basic Information of Ten Herbal Injections in Cancer Care

The basic information of ten herbal injections involved in the national essential health insurance program is listed in Table 1, Supplementary Table S1. According to the classification of the health insurance catalog, six injections were defined as ZC01 anti-cancer drugs, and four were ZC02 adjuvant drugs for cancer. Four injections contained more than one herbal composition, while six injections contained a single compound or ingredients extracted from a single herb as the main component. The health insurance has made the following restrictions on the payment scope of each injection. All injections were restricted to be used in secondary and tertiary hospitals, except for two injections, Cinobufacin and *Polyporus* polysaccharide.

**TABLE 1:** The basic information for ten Chinese herbal injections for cancer covered in the National Essential Health Insurance Program.

Name	Approved year	Main components	Actions and Indications	Dosage	Insurance coverage
Compound Kushen injection (ZC01 anti-cancer drug)	1988	Extracts from Sophora flavescens Aiton (Kushen), Smilax glabra Roxb. (Baituling)	Actions: Clearing heat and draining dampness, cooling the blood and resolving toxin, dissipating binds and relieving pain; Indications: Cancer pain and bleeding	Intramuscular injection, 2∼4 ml once, 2 times a day; or intravenous drip, 20 ml once, diluted with 200 ml sodium chloride injection and applied, once a day, children should reduce it, the total amount of systemic medicine 200 ml is a course of treatment, generally it can be used continuously for 2 to 3 courses; or as directed by a doctor	Limited to be used for middle and advanced cancers in secondary and tertiary hospitals
Shenqi Fuzheng injection (ZC02 adjuvant treatment for cancer)	1999	Extracts from Codonopsis pilosula (Franch.) Nannf. (Dangshen)*,* Astragalus: Astragalus membranaceus (Fisch.) Bunge. (Huangqi)	Action: Boosting qi and reinforcing the healthy qi; Indications: Fatigue, asthenic breathing, laziness to speak, spontaneous sweating and dizziness caused by lung-spleen-qi deficiency; adjuvant treatment for lung cancer and gastric cancer with the above symptoms	Intravenous drip. 250 ml each time (i.e., 1 bottle), once a day, treatment for 21 days; combined with chemotherapy, start to use 3 days before chemotherapy, and the course of treatment can end simultaneously with chemotherapy	Limited to be used in secondary and tertiary hospitals, concurrent use with radiotherapy and chemotherapy for lung cancer and gastric cancer together with evidence of blood indicators and low immune function
Aidi Injection (ZC01 anti-cancer drug)	1996	Astragalus: Astragalus membranaceus (Fisch.) Bunge. (Huangqi), Eleutherococcus senticosus: Acanthopanax senticosus (Rupr. Maxim.) Harms (Ciwujia), Ginseng: Panax ginseng C. A. Mey. (Renshen), Mylabris phalerata (Pallas) (Banmao)	Actions: Clearing heat and removing toxin, eliminating blood stasis and dissipating binds; Indications: Primary liver cancer, lung cancer, rectal cancer, malignant lymphoma, gynecological malignancyetc.	Intravenous drip. Adults 50∼100 ml once, add 0.9% sodium chloride injection or 5%∼10% glucose injection 400∼450 ml, once a day; when combined with radiotherapy and chemotherapy, the course of treatment is synchronized with radiotherapy and chemotherapy; use before and after surgery 10 days of this product is a course of treatment; 10 days of interventional treatment is a course of treatment; 15 days of single use is a cycle, with an interval of 3 days, and 2 cycles are a course of treatment; for patients with advanced cachexia, 30 days of continuous use is a course of treatment, or depending on the condition	Limited to be used for middle and advanced cancers in secondary and tertiary hospitals
Kangai injection (ZC02 adjuvant treatment for cancer)	2002	Astragalus: Astragalus membranaceus (Fisch.) Bunge. (Huangqi), Ginseng: Panax ginseng C. A. Mey. (Renshen), Ammothamnine	Actions: Boosting qi and reinforcing healthy qi, strengthening the body's immune function; Indications: Primary liver cancer, lung cancer, rectal cancer, malignant lymphoma, gynecological malignant tumors; Leukopenia and hypoxia caused by various reasons; Chronic hepatitis B	Slow intravenous injection or drip; 1–2 times a day, 40–60 ml a day, diluted with 5% glucose or 0.9% saline 250–500 ml before use. 30 days is a course of treatment or as prescribed by doctor	Limited to be used for middle and advanced malignant tumors indicated in the instructions of secondary and tertiary hospitals
Kanglaite injection (ZC01 anti-cancer drug)	1997	Oil from Coix lacryma-jobi L. for injection (Oil from Yiyiren)	Actions: Boosting qi and nourishing yin, eliminating mass and dissipating binds Indications: Primary non-small cell lung cancer and primary liver cancer with qi-yin deficiency pattern or spleen deficiency and dampness encumbrance pattern; It has a certain synergistic effect with radiotherapy and chemotherapy; It has certain anti-cachexia and analgesic effects for patients the medium and advanced tumors	Slowly inject 200 ml intravenously, once a day, for 21 days as a course of treatment, and the next course of treatment can be performed after an interval of 3–5 days. When combined with radiotherapy and chemotherapy, the dose can be reduced. For the first use, the drip rate should be slow; the drip rate should be 20 drops/min in the first 10 min, it can continue to increase after 20 min, and it can be controlled at 40–60 drops/min after 30 min	Limited to be used for middle and advanced lung or liver cancers in secondary and tertiary hospitals
Xiaoaiping injection (ZC01 anti-cancer drug)	2002	Marsdenia tenacissima (Roxb.) Moon (Tongguanteng)	Actions: Clearing heat and removing toxin, resolving phlegm and softening hardness; Indications: It is used for esophageal cancer, gastric cancer, lung cancer, liver cancer, and can be used as adjuvant treatment for radiotherapy and chemotherapy	Intramuscular injection: 2∼4 ml once, 1∼2 times a day; or as directed by a doctor. Intravenous drip: Dilute with 5% or 10% glucose injection and instill infusion, 20∼100 ml once, once a day; or follow the doctor's advice	Limited to be used for middle and advanced cancers in secondary and tertiary hospitals
Cinobufacin injection (ZC01 anti-cancer drug)	2001	Cinobufacin	Actions: Removing toxin, dispersing swelling and relieving pain; Indications: Middle and advanced tumors, chronic hepatitis B and other diseases	Intramuscular injection, 2∼4 ml once (2/5∼4/5), 2 times a day; intravenous drip, once a day, 10∼20 ml (2∼4) once, injected with 5% glucose Dilute 500 ml of the solution and instill it slowly, take the medicine for 7 days, rest for 1 to 2 days, four weeks as a course of treatment, or follow the doctor's advice	Limited to be used for patients with cancer pain and difficulty swallowing
*Brucea javanica* oil emulsion injection (ZC01 anti-cancer drug)	1994	Oil emulsion from *Brucea javanica* (L.) Merr. (Yadanzi)	Actions: Anti-cancer drug Indications: Lung cancer, lung cancer brain metastases and digestive tract tumors	Intravenous drip, 10∼30 ml once (1∼3 tubes at a time), once a day (this product must be added with 250 ml of sterile normal saline, use immediately after dilution)	Limited to be used for middle and advanced cancers in secondary and tertiary hospitals
*Polyporus* polysaccharide injection (ZC02 adjuvant treatment for cancer)	1999	*Polyporus* polysaccharide	Indications: It can regulate the immune function of the body and has a certain effect on chronic hepatitis and tumors. Combined with anti-tumor chemotherapy drugs, it can enhance the efficacy and reduce toxic side effects	Intramuscular injection. 2∼4 ml once, once a day, the children should reduce it or follow the doctor's advice	Limited to be used for patients with low immune function during chemotherapy of malignant tumor
*Astragalus* polysaccharide for injection (ZC02 adjuvant treatment for cancer) (in the negotiation list during the agreement period)	2001	*Astragalus* polysaccharide	Supplement qi deficiency. It can be used to treat cancer patient with leukopenia, impaired immunity and low level of quality of life after chemotherapy, which shows symptoms like fatigue, spontaneous sweating, short of breath, and poor appetite due to qi deficiency	Intravenous drip, use a syringe to draw 10 ml saline into the vial, shake immediately until the medicine is completely dissolved, then add it to 500 ml 0.9% sodium chloride injection or 5–10% glucose injection, drip. The time is not less than 2.5 h 250 mg once, once a day. The course of treatment is 21 days for patients with immunocompromised function and 7 days for other treatment courses	Limited to be used in secondary and tertiary hospitals. A single hospitalization can be paid for up to 14 days

Note: ZC, the drug classification code, Z for Chinese patent herbal drugs and C for cancer medication. Approved year refers to the earliest time to market according to the approved number for each injection.

### The Selection of RCTs, Systematic Reviews and Meta-analyses

**FIGURE 1:**
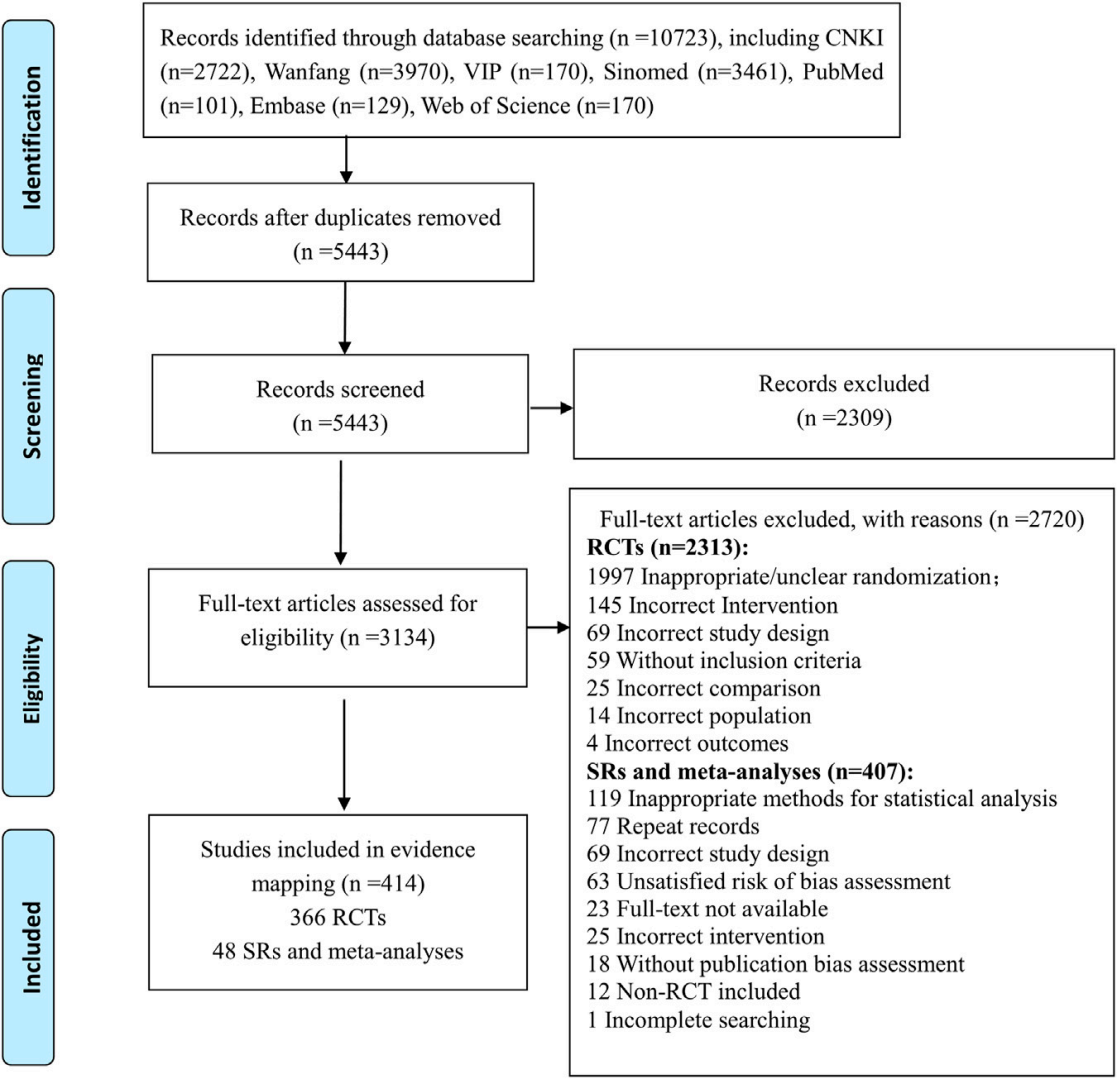
Flow Diagram of the selection of RCTs, systematic reviews, and meta-analyses.

The flow diagram of the literature selection is shown in Figure 1. A total of 10,723 records were identified from the initial search through seven databases. After removal of duplicates and exclusion of references by reading titles and abstracts, 3134 full-text articles were screened and 2720 references were excluded with reasons (seen in the flow chart). Finally, 414 studies composed of 366 RCTs and 48 SRs and meta-analyses were included in this evidence mapping.

The numbers of included studies of the ten herbal injections are shown in Table 2. The top three injections in the included RCTs were Compound Kushen (78, 21.3%), Shenqi Fuzheng (76, 20.8%), and Aidi injection (63, 17.2%), and the top three injections in the SRs were Kanglaite (10, 20.8%), Compound Kushen (9, 18.8%), and Shenqi Fuzheng injection (8, 16.7%). No eligible evidence was identified for *Polyporus* polysaccharide injection. The remaining analysis only includes nine injections.

**TABLE 2:** Number of included studies on ten Chinese herbal injections used in cancer care.

Name of the Chinese herbal injections	No. of RCTs (%)	No. of SRs/meta-analyses (%)
Compound Kushen injection	78 (21.3)	9 (18.8)
Shenqi fuzheng injection	76 (20.8)	8 (16.7)
Aidi injection	63 (17.2)	5 (10.4)
Kangai injection	45 (11.7)	4 (8.3)
Kanglaite injection	36 (9.8)	10 (20.8)
Xiaoaiping injection	25 (6.8)	2 (4.2)
Cinobufacin injection	25 (6.8)	5 (10.4)
*Brucea javanica* oil emulsion injection	13 (3.6)	5 (10.4)
*Polyporus* polysaccharide injection	0 (0)	0 (0)
*Astragalus* polysaccharide for injection	5 (1.37)	0 (0)
Total	366 (100)	48 (100)

Note: RCTs, Randomized controlled trials; SRs, systematic reviews and meta-analyses.

### Bibliometric Information and Characteristics of Included RCTs

**FIGURE 2:**
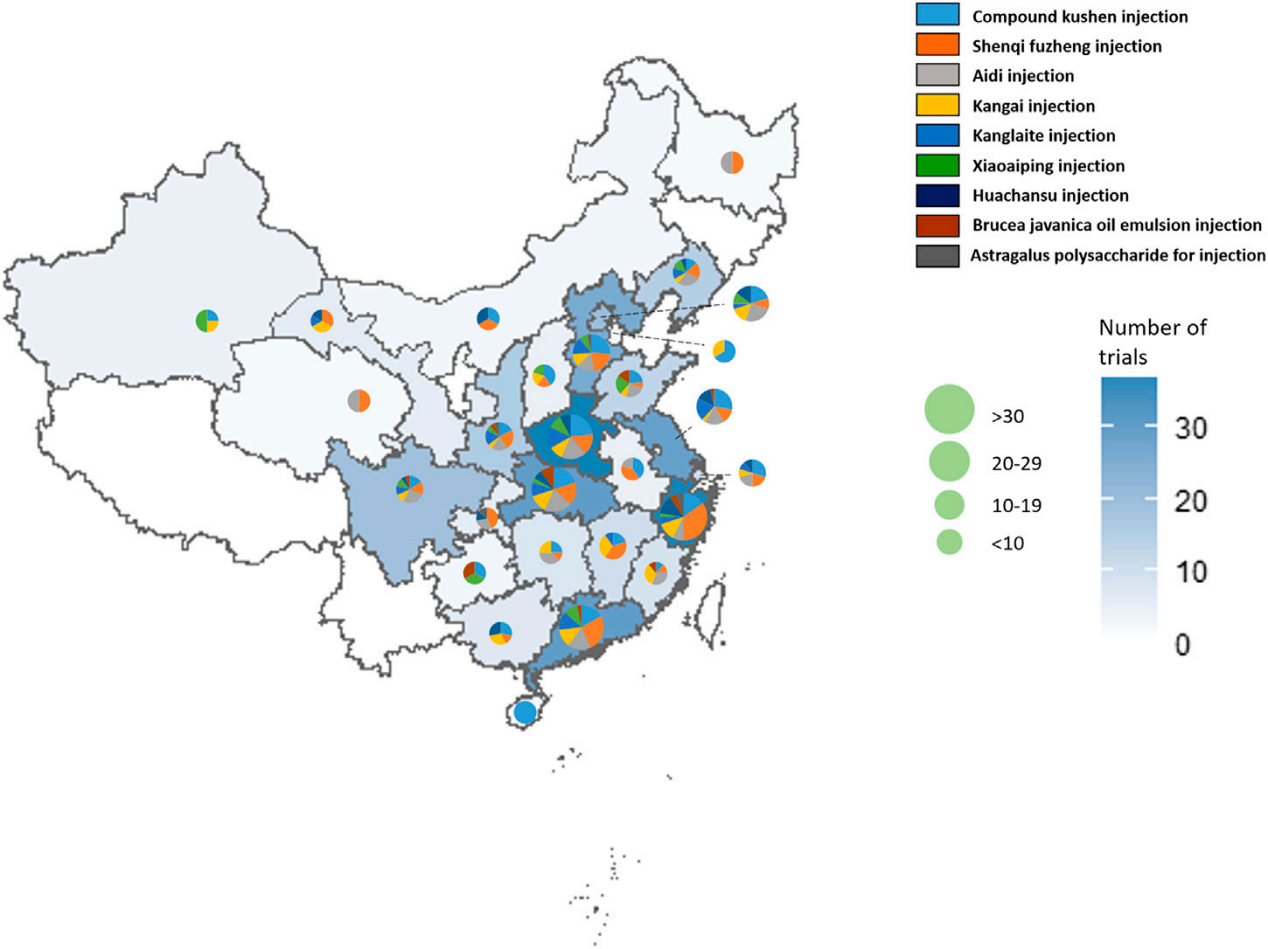
The geographical distribution of the primary investigators of RCTs on herbal injections for cancer care.

In Table 3, the increasing overall trend in the number of studies is demonstrated. Before 2009, trials were conducted sporadically. The number increased rapidly after 2009 before plateauing from 2015, with about 40 to 60 newly published trials each year. Geographical distribution of RCTs of nine herbal injections for which there was eligible evidence was uneven according to Figure 2. Published studies were located in 27 different provinces and municipalities. The largest number of RCTs were from Henan province (n = 39), while no eligible published RCTs were found in Jilin Province, Ningxia Hui Autonomous Region, Tibet Autonomous Region, and Yunnan Province. In Zhejiang Province, there were 14 studies on Shenqi Fuzheng injection, the largest number of studies for one injection in a single province.

**TABLE 3:** The numbers of RCTs on ten herbal injections used in cancer care.

Name of the Chinese herbal injections	2003	2006	2007	2008	2009	2010	2011	2012	2013	2014	2015	2016	2017	2018	2019	2020
Compound Kushen injection	0	1	0	1	0	3	5	3	5	3	10	10	7	10	13	7
Shenqi Fuzheng injection	0	1	4	2	1	1	0	2	5	8	8	4	9	14	11	6
Aidi injection	0	1	0	0	4	2	1	2	1	7	8	11	8	8	5	5
Kangai injection	0	0	0	2	2	1	2	1	2	7	1	4	8	10	3	2
Kanglaite injection	1	1	0	0	0	0	2	1	2	0	6	3	3	9	7	1
Xiaoaiping injection	0	0	0	0	0	0	0	1	1	1	5	3	4	2	6	2
Cinobufacin injection	0	0	0	0	1	0	1	1	1	5	3	2	4	4	2	1
*Brucea javanica* oil emulsion injection	0	0	0	0	1	0	1	0	0	0	2	1	1	3	3	1
*Astragalus* polysaccharides for injection	0	0	0	0	1	0	0	0	0	1	0	1	1	0	0	1
Total	1	4	4	5	10	7	12	11	17	32	43	39	45	60	50	26

The included RCTs involved a total of 31,233 cancer participants, and the sample sizes of individual studies mostly ranged from 51–100 (265, 72.4%) (Table 4). The included studies involved 15 types of cancers and multiple cancer types, although lung cancer was the most frequently studied type of cancer (118, 32.2%). Only 26 studies mentioned TCM syndrome, accounting for 7.1%. Of the RCTs, 97.0% used injections with conventional treatment as combined therapy. With regard to the course of treatment of herbal injection, the injections and standard therapy were implemented simultaneously in 297 studies. Among them, the injections started and ended at the same time as the conventional therapy for each course in 157 studies (42.9%), and the other 140 studies (38.3%) had the injections end early before the end of conventional treatment. For the controls, the included RCTs mainly involved 13 types of controls, among which palliative chemotherapy was used most commonly (46.4%). As for the methodological characteristics of the included RCTs, 341 RCTs (93.2%) were single-center trials, and blinding was implemented only in four trials (1.1%). A total of 92 RCTs reported funding sources, of which 82 received government funding (22.4%), seven university/hospital funding (1.9%), and three received other support.

**TABLE 4:** The clinical and methodological characteristics reported in the included RCTs of Chinese herbal injections for cancer care.

Items	Details	N (%)
Study setting	Single-center study	341 (93.2)
	Multicenter study	17 (4.6)
	Not reported	8 (2.2)
Sample size	≤50	33 (9.0)
	51–100	265 (72.4)
	101–200	60 (16.4)
	＞200	8 (2.2)
Populations	Lung cancer	118 (32.2)
	Colorectal cancer	39 (10.7)
	Stomach cancer	39 (10.7)
	Liver cancer	32 (8.7)
	Breast cancer	28 (7.7)
	Hematological malignancy	19 (5.2)
	Esophagus cancer	14 (3.8)
	Cervical cancer	11 (3.0)
	Nasopharyngeal cancer	10 (2.7)
	Ovarian cancer	8 (2.2)
	Pancreatic cancer	8 (2.2)
	Prostate cancer	4 (1.1)
	Bladder cancer	4 (1.1)
	Renal cancer	1 (0.3)
	Laryngeal cancer	1 (0.3)
	Multiple cancer	30 (8.2)
TCM syndrome	Reported	26 (7.1)
	Not reported	340 (92.9)
Interventions	Injection used as add-on treatment	355 (97.0)
	Injection only	11 (3.0)
Time of administration of herbal injections	Start and end at the same time as the standard therapy	157 (42.9)
	Start at the same time as the standard therapy, but end earlier	140 (38.3)
	Start before the standard therapy, but end at the same time as standard therapy	19 (5.2)
	Start at the same time as the standard therapy, but end later	16 (4.4)
	Start before the standard therapy, and end before the standard therapy	7 (1.9)
	Start after the standard therapy	3 (0.8)
	Start before the standard therapy and end earlier	2 (0.5)
	Used alone	5 (1.4)
	Others^a^	17 (4.6)
Administration of herbal injections	i.v.gtt	335 (91.5)
	i.m	4 (1.1)
	i.p	8 (2.2)
	Thoracic perfusion	5 (1.4)
	Arterial infusion	2 (0.5)
	p.r	2 (0.5)
	Multiple administrations	3 (0.8)
	Not reported	7 (1.9)
Controls	Palliative chemotherapy	170 (46.4)
	Adjuvant chemotherapy	28 (7.7)
	Chemo/radiotherapy	24 (6.6)
	Radiotherapy	21 (5.7)
	Curative chemotherapy (tumors of the blood, lymphatic and reproductive system)	19 (5.2)
	Supportive/symptomatic treatment	16 (4.4)
	Interventional therapy^a^	14 (3.8)
	Chemotherapy (Unable to identify the type)	11 (3.0)
	Perfusion therapy (chemotherapy infused into the chest or abdomen)	11 (3.0)
	Neoadjuvant chemotherapy	9 (2.5)
	Targeted therapy	8 (2.2)
	Placebo	2 (0.5)
	Placebo with XELOX chemotherapy	1 (0.3)
	Multiple controls	1 (0.3)
	Others^a^	31 (8.5)
Random sequence generation	Random number tables	339 (92.6)
	Draw lots	17 (4.6)
	Computer-generated random sequences	4 (1.1)
	Toss a coin	3 (0.8)
	Random envelopes	3 (0.8)
Blinding	Blinding	4 (1.1)
	No blinding	362 (98.9)
Funding source	Government	82 (22.4)
	University/hospital	7 (1.9)
	Multiple funding	1 (0.3)
	Charity fund	1 (0.3)
	Medical association	1 (0.3)
	Not reported	274 (74.9)

Notes: i.v.gtt, intravenous drip; i.m., intramuscular injection; i.p., intraperitoneal injection; p.r., rectal administration.

Interventional therapy: including transarterial chemoembolization (TACE) and radiofrequency ablation.

a Other timing of administration of Chinese herbal injection including used before control, or started days before the surgery and stopped days after the surgery, or used for 21 days continuously and stopped for 7 days without relation to the control, etc.Other controls including Interleukin infused into the abdomen, analgesia therapy, adjuvant chemotherapy combined with palliative chemotherapy, antiviral therapy, perfusion therapy into bladder, etc.

**FIGURE 3:**
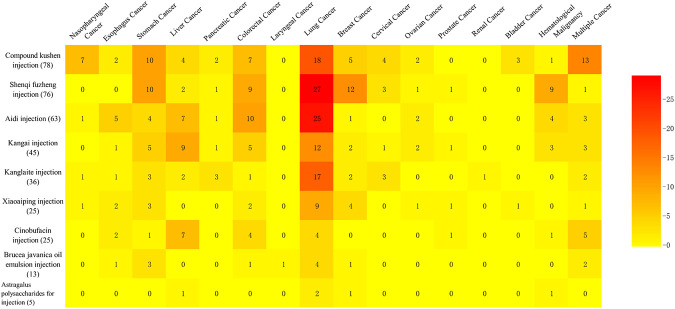
Evidence mapping of the distribution of nine herbal injections by cancer type.

Of 366 studies, 336 RCTs studied the use of an injection to treat a certain type of cancer and involved 15 different kinds of cancer, while the remaining 30 RCTs studied the use of a specific injection for multiple cancers (Figure 3). Lung cancer was the most studied type of cancer, involving 118 RCTs on nine types of injection, followed by colorectal cancer (39 studies) and stomach cancer (39 studies), which both covered eight herbal injections. In addition, Compound Kushen injection was tested on a wide range of types of cancers (12 types).

### Clinical Characteristics of the Included Systematic Reviews and Meta-analyses

As shown in Table 5, the included 48 SRs involved a total of 65,884 participants, of which the sample size per review ranged from 61 to 3433, mostly distributed in ≤1000 (23 SRs, 47.9%). Seven types of cancer and multiple cancer syndromes were evaluated in the SRs while lung cancer (20 SRs, 41.7%), liver cancer (6 SRs, 12.5%), stomach cancer (5 SRs, 10.4%), and colorectal cancer (5 SRs, 10.4%) were the most reviewed cancers. It is worth mentioning that the injections were only used as an add-on treatment in all of the 48 SRs. In addition, there were six kinds of controls mentioned in the SRs while 35 SRs (72.9%) included chemotherapy as the comparator.

**TABLE 5:** The clinical characteristics of the included systematic reviews and meta-analyses of Chinese herbal injections for cancer care.

Items	Details	N (%)
Sample size	≤1000	23 (47.9)
	1001–2000	11 (22.9)
	2001–3000	11 (22.9)
	＞3000	3 (6.3)
Populations	Lung cancer	20 (41.7)
	Liver cancer	6 (12.5)
	Colorectal cancer	5 (10.4)
	Stomach cancer	5 (10.4)
	Breast cancer	3 (6.3)
	Esophagus cancer	3 (6.3)
	Hematological malignancy	1 (2.1)
	Multiple cancer	5 (10.4)
Interventions	Injection used as add-on treatment	48 (100.0)
Administration of herbal injections	Intravenous drip	5 (10.4)
	Multiple routes of administrations	2 (4.2)
	Not reported	41 (85.4)
Controls	Chemotherapy	35 (72.9)
	Transarterial chemoembolization (TACE)	6 (12.5)
	Targeting therapy	2 (4.2)
	Others	5 (10.4)

Other controls included radiotherapy, radiotherapy alone or chemoradiotherapy, radiotherapy and conventional therapy for cancer pain, chemo or radiotherapy, and chemotherapy or chemotherapy with conventional therapy.

### Outcomes and Effects of the Herbal Injections

**FIGURE 4:**
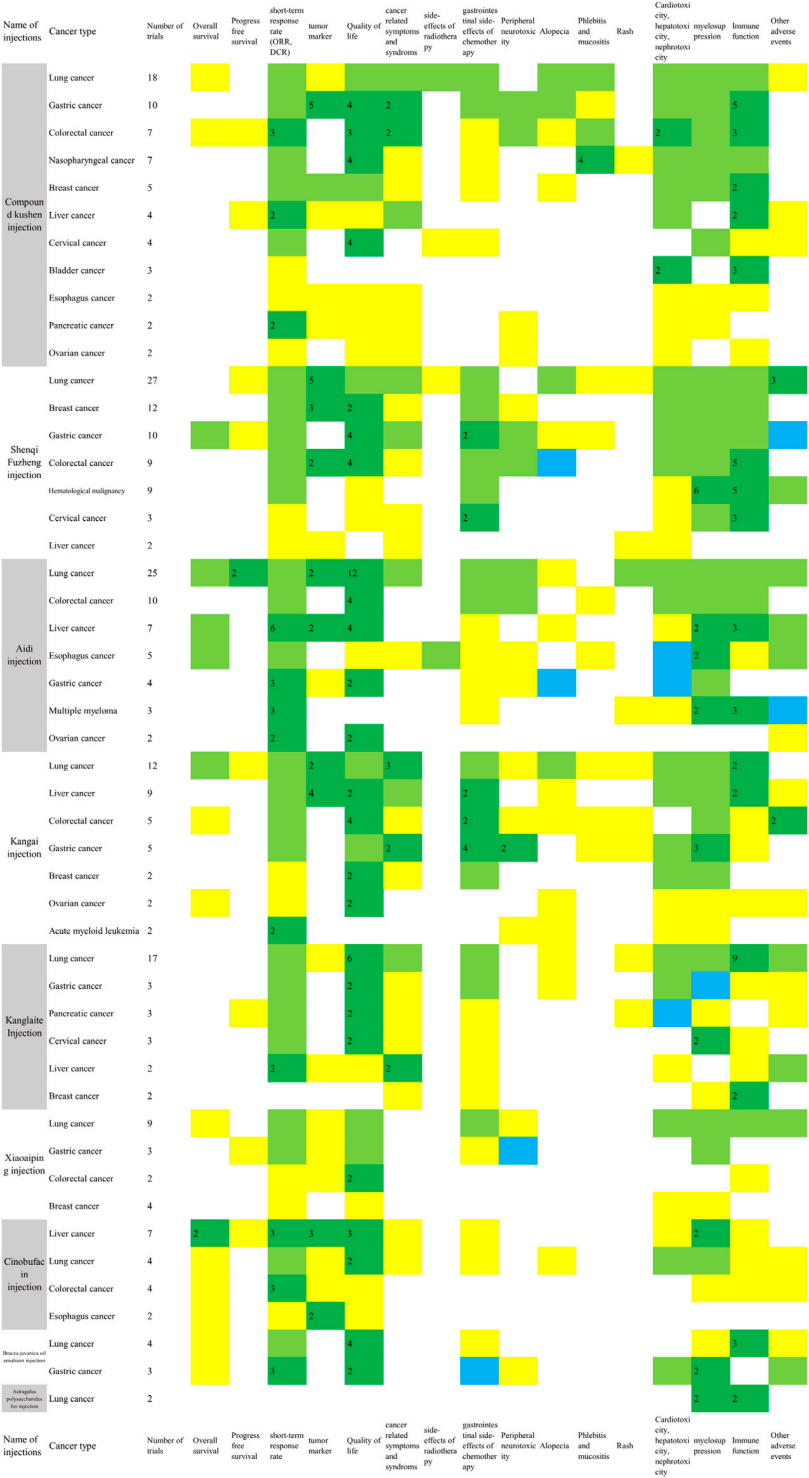
Evidence mapping of the reported clinical outcomes by cancer types (Two or more RCTs for a single cancer type were shown in Figure 4. Dark green: at least two trials showed consistent beneficial effect for all measurements on summarized outcome with no significant or harmful effects; Light green: some of the reported outcomes showed beneficial effect and some did not show significant effect; Yellow: a single study reported the outcome in spite of the direction of the effect. Blue: all of the reported outcomes showed no significant effects; Red: any study showed harmful effects. The number in dark green cells presented the number of RCTs. ORR, objective response rate; DCR, disease control rate; Quality of life includes KPS scores, ECOG scores and other scales for quality of life; Cancer related symptoms include fatigue, cancer pain, cancer fever and decrease of weight; Side-effects of radiotherapy include radiation pneumonitis, radiation esophagitis and radiation enteritis; Gastrointestinal side-effects of chemotherapy include nausea and vomiting, diarrhea, and constipation; myelosuppression includes leukopenia, neutropenia, thrombocytopenia, anemia, and erythrocytopenia; Immune function includes CD3, CD4, CD4/CD8, and NK cell level.).

The metrics of clinical outcomes and cancer types is shown in Figure 4. The consistent beneficial effect (at least two trials showed beneficial effect with no significant or harmful effects) according to the reported RCTs are shown in dark green, and the numbers of RCTs for each outcome are written in the cells. The outcomes of included SRs are presented in Supplementary Table S2.

#### Compound Kushen Injection

In the 78 RCTs for Compound Kushen injection, 11 cancer types were included, with more than two trials for each type, and it was commonly used for lung cancer (18, 23.1%), gastric cancer (10, 12.8%), colorectal cancer (7, 9.0%), and nasopharyngeal cancer (7, 9.0%). Compared with conventional therapy alone, the combined treatment with Compound Kushen injection showed consistent beneficial effects on objective response rate (ORR) for colorectal cancer (3 RCTs) and gastric cancer (2 RCTs), or a disease control rate (DCR) for breast cancer (2 RCTs), and pancreatic cancer (2 RCTs). It is worth mentioning that adding in Compound Kushen injection showed consistent effect on pneumonitis caused by radiation treatment in lung cancer patients (4 RCTs) while all other injections had insufficient consistent benefit on radiation pneumonitis. The myelosuppression and immune function were also shown to be improved in several cancer types based on 2-6 RCTs (Figure 4).

In the nine SRs concerning Compound Kushen injection, three SRs were for advanced non-small cell lung cancer (NSCLC) patients. Compared with cisplatin-based chemotherapy alone, Compound Kushen injection showed beneficial effects for advanced NSCLC patients on ORR, KPS scores, leukopenia in 3 SRs, one-year survival rate, DCR, cancer pain relief, nausea and vomiting, diarrhea, alopecia, mucositis, thrombocytopenia, and anemia in one SR (Supplementary Table S2).

#### Shenqi Fuzheng Injection

In the 76 RCTs involving Shenqi Fuzheng injection, only one trial tested Shenqi Fuzheng injection for immune function in lung cancer patients during perioperative period compared with normal saline, while other trials tested the add-on effect of Shenqi Fuzheng injection. In terms of cancer types, 27 (35.5%) of the trials examined Shenqi Fuzheng injection in lung cancer patients, followed by breast cancer (12, 15.8%) and gastric cancer (10, 13.2%). Among the survival and tumor responses, only ORR for colorectal cancer showed consistent beneficial effect (3 RCTs). Improved quality of life was found in breast cancer (2 RCTs), gastric cancer (4 RCTs), and colorectal cancer (4 RCTs), and relief of cancer fatigue was found in both lung (5 RCTs) and gastric cancer patients (2 RCTs) with consistency. Add-on therapy with Shenqi Fuzheng injection showed obvious consistent effect in immune function in 3-5 trials (Figure 4).

In the eight SRs concerning Shenqi Fuzheng injection, three SRs were for advanced NSCLC patients. Compared with cisplatin based chemotherapy alone, Shenqi Fuzheng injection combined with NP (navelbine and cisplatin), GP (gemcitabine and cisplatin), and TP (paclitaxel and cisplatin) regimen showed beneficial effects on ORR (2 SRs), KPS scores (3 SRs), cancer fatigue (1 SR), cancer fever (2 SRs), leukopenia (3 SRs), thrombocytopenia (3 SRs), anemia (2 SRs), and immune function assessed by CD4, CD4/CD8, and NK cells level (2 SRs). In terms of breast cancer (2 SRs), compared with chemotherapy alone, add-on with Shenqi Fuzheng injection showed beneficial effects on ORR, KPS scores, alopecia, liver and kidney function, leukopenia, thrombocytopenia, CD4, CD4/CD8, and abnormal changes in electrocardiogram in two SRs, and reducing cancer fatigue, nausea and vomiting, CD3, and NK cells level in one SR (Supplementary Table S2).

#### Aidi Injection

Twenty-five out of 63 RCTs tested the effects of Aidi injection for lung cancer. Compared with chemo or targeting therapy alone, add-on with Aidi injection showed consistent effects on three-year survival rate (2 RCTs), progression free survival (2 RCTs), KPS scores (12 RCTs), and immune function assessed by CD4 and CD4/CD8 (10 RCTs) in lung cancer patients (Figure 4). The add-on with Aidi injection showed beneficial effects on ORR (2 SRs), DCR (1 SR), KPS scores improvement (2 SRs), neutropenia (1 SR), leukopenia (1 SR), thrombocytopenia (2 SRs), nausea and vomiting (1 SR), and liver function (1 SR) compared to chemotherapy alone in advanced NSCLC patients (Supplementary Table S2).

Compared with chemotherapy alone, add-on with Aidi injection showed significant effects on ORR (1 SR), DCR (1 SR), KPS scores (2 SRs), nausea and vomiting (1 SR), peripheral neurotoxicity (1 SR), diarrhea (1 SR), immune function (1 SR) assessed by CD3, CD4, CD4/CD8, and NK cells level, and leukopenia (2 SRs) in colorectal cancer patients. In one SR on liver cancer, it showed beneficial effects of add-on with Aidi injection on ORR, KPS scores, survival rate, and immune function assessed by CD3 and CD4 compared to transarterial chemoembolization (TACE) treatment alone. Compared with conventional therapy alone, the add-on with Aidi injection showed consistent benefits on ORR in liver cancer patients (6 RCTs), gastric cancer (3 RCTs), multiple myeloma (3 RCTs), and ovarian cancer (2 RCTs). The improvement of KPS scores were also observed in colorectal cancer (4 RCTs), gastric cancer (2 RCTs), and ovarian cancer (2 RCTs) (Figure 4).

#### Kangai Injection

Only one RCT compared Kangai injection with placebo for advanced liver cancer; others tested combined effects of Kangai injection and conventional therapy. Twelve studies were focused on lung cancer. Compared with chemotherapy alone in 10 RCTs, the add-on with Kangai injection showed significant effects on DCR (3 RCTs), quality of life assessed by SF-36 (2 RCTs), cancer fatigue (3 RCTs), tumor marker (2 RCTs), and leukopenia (3 RCTs) in lung cancer patients. In liver cancer trials, the add-on with Kangai injection showed effects on KPS scores (5 RCTs) and improvement in liver function (7 RCTs) compared with conventional therapy. In two SRs for liver cancer, the combination of Kangai injection and TACE showed improvement on KPS scores, cancer pain, leukopenia, anemia, and liver function (Supplementary Table S2).

Other significant outcomes demonstrated ORR, KPS scores improvement, neurotoxicity, and leukopenia in colorectal (1 SR) and gastric cancer patients (1 SR), KPS scores improvement in colorectal cancer patients (4 RCTs), and relieving nausea and vomiting in gastric cancer patients (4 RCTs) (Figure 4).

#### Kanglaite Injection

Seventeen of the 36 included RCTs tested Kanglaite injection for lung cancer. Compared with palliative chemotherapy alone, the add-on with Kanglaite improved quality of life assessed by KPS scores (6 RCTs) and EORTC-QLQ-C30 (5 RCTs), and the immune function assessed by CD3 (7 RCTs), CD4 (8 RCTs), and CD4/CD8 (6 RCTs). Compared with GP chemotherapy alone, the add-on with Kanglaite reduced cancer pain in advanced NSCLC patients (2 RCTs) (Figure 4). Eight out of 10 included SRs tested Kanglaite for lung cancer. Compared with chemotherapy alone, Kanglaite combined with chemotherapy showed significant effects on one-year survival rate (2 SRs), ORR (6 SRs), DCR (4 SRs), improved KPS scores (6 SRs), weight improvement (1 SR), nausea and vomiting (4 SRs), leukopenia (4 SRs), thrombopenia (3 SRs), anemia (1 SR), improvement on CD3 level (2 SRs), CD4 level (3 SRs), CD4/CD8 (2 SRs), and NK cells level (1 SR) in lung cancer patients. Compared with gefitinib alone, the add-on with Kanglaite improved KPS scores in lung cancer patients (2 SRs) (Supplementary Table S2).

In addition, combining Kanglaite with conventional treatment also showed beneficial effects on ORR and reducing cancer fever in liver cancer patients (2 RCTs), reducing nausea and vomiting (2 RCTs), leukopenia (1 SR), improving ORR (1 SR) in gastric cancer patients, and improving KPS scores (2 RCTs) and leukopenia in cervical cancer patients (2 RCTs) (Figure 4).

#### Xiaoaiping Injection

Nine out of 25 RCTs tested the add-on effect of Xiaoaiping injection for lung cancer. Compared to palliative platinum-based chemotherapy alone, the combined treatment with Xiaoaiping for 14 to 21 days in one chemotherapy cycle for 2-4 cycles showed beneficial effects on KPS scores (6 RCTs), improvement of white blood cell reduction (6 RCTs), and CD4 and CD4/CD8 level (3 RCTs) in IIIB-IV advanced lung cancer patients. The evidence mapping also showed that compared with chemotherapy alone, palliative chemotherapy with oxaliplatin and Xiaoaiping (intravenous infusion or enema) improved KPS scores for colorectal cancer (2 RCTs). No consistent beneficial effect was observed in the included RCTs in breast and gastric cancer patients (Figure 4). Compared to TACE alone, Xiaoaiping plus TACE showed significant effects on ORR and KPS scores in liver cancer patients (1 SR). Compared with chemotherapy alone, Xiaoaiping plus chemotherapy showed significant effects on improving appetite, dysphagia, cancer pain, and KPS scores in esophageal cancer patients (1 SR) (Supplementary Table S2).

#### Cinobufacin Injection

Seven out of 25 RCTs of Cinobufacin injection were for liver cancer. Compared with radiotherapy or TACE therapy alone, the add-on with intravenous or artery perfusion of Cinobufacin showed consistent effects on one-year survival rate (2 RCTs and 1 SR), two-year survival rate (1 SR), KPS scores (1 RCT and 1 SR), ORR (1 RCT and 1 SR), DCR (1 RCT), and tumor marker (3 RCTs) in liver cancer. Compared with supportive care alone, the combination with intravenous or intraperitoneal perfusion of Cinobufacin showed a beneficial effect on improved KPS scores in two trials for liver cancer. In terms of lung cancer, the add-on with Cinobufacin to chemotherapy showed significant effects on one-year survival rate (1 SR), two-year survival rate (2 SRs), improved KPS scores (2 SRs), ORR (2 SRs), relief for cancer pain (2 SRs), nausea and vomiting (1 SR), leukopenia reduction (1 SR), and thrombocytopenia reduction (2 SRs), but increased phlebitis occurrence, which was the only harmful outcome identified in one SR (Supplementary Table S2).

In addition, the add-on with Cinobufacin to chemo/radiotherapy also showed beneficial effect on ORR for colorectal cancer (3 RCTs) (Figure 4). In esophageal cancer trials, add-on with Cinobufacin showed significant effect on one- and two-year survival rate compared with radiotherapy (1 SR). Cinobufacin combined with chemotherapy was also associated with better effects than chemotherapy alone in improving ORR and KPS scores in gastric cancer (1 SR).

#### *Brucea javanica* Oil Emulsion Injection

The evidence mapping showed beneficial effects for lung cancer and gastric cancer in RCTs combined with *Brucea javanica* oil emulsion injection. Compared with palliative platinum-based chemotherapy or gefitinib alone, add-on with *Brucea javanica* oil emulsion showed beneficial effects on KPS score and immune function including CD3, CD4, and CD4/CD8 level in lung cancer patients (3 RCTs). Compared with palliative chemotherapy with oxaliplatin or paclitaxel alone, adding *Brucea javanica* oil emulsion showed beneficial effects on ORR (3 RCTs) and DCR (2 RCTs) in gastric cancer patients (Figure 4). Compared with chemotherapy alone, *Brucea javanica* oil emulsion showed significant effects on ORR, improved quality of life, and reduced leukopenia events in lung cancer (2 SRs), and advanced colorectal cancer (1 SR). One SR found that add-on with *Brucea javanica* oil emulsion significantly improved one-year and two-year survival rate and ORR compared to radiotherapy alone in esophagus cancer (Supplementary Table S2).

#### *Astragalus* Polysaccharide for Injection

Two RCTs using *Astragalus* polysaccharide for injections were identified for the treatment of lung cancer. The combination of *Astragalus* polysaccharide for injections and radiotherapy (1 RCT) or palliative chemotherapy (1 RCT) improved the leukopenia and increased the level of CD3, CD4, and CD4/CD8.

## Discussion

### Summary of Findings

This evidence mapping for Chinese herbal injection in cancer care was based on 366 RCTs and 48 SRs and meta-analyses and provided a broad overview on available evidence of herbal injections and the effects on outcomes in different cancer types. More than half of the RCTs were for Compound Kushen, Shenqi Fuzheng, and Aidi injection, and nearly one-third of the diseases in RCTs were lung cancer, followed by colorectal cancer and gastric cancer. The majority of the RCTs and all SRs examined the add-on effect of herbal injections.

The outcome and effect metric showed generally similar applications in herbal injections in cancer care. More consistent benefits were observed in Compound Kushen, Shenqi Fuzheng, Aidi, and Kangai injections for tumor response, quality of life, myelosuppression, and immune function improvement. The survival outcomes were only reported consistently for Aidi and Cinobufacin injections based on four individual RCTs. The most reported outcomes of relieving side-effects of the standard therapy were nausea and vomiting, white blood cells, CD4 levels, and CD4/CD8.

### Differences From Previous Studies

The use of mapping to provide an evidence summary has been used in the Chinese medicine field for Chinese herbs for hypertension (Wei et al., 2019), Taiji (Solloway et al., 2016), and massage (Miake-Lye et al., 2019), but there has been no evidence mapping report on Chinese herbal injections. Most of the articles published in English on Chinese herbal injections were literature reviews (Wang et al., 2018), mainly introducing the biological mechanisms or network meta-analyses (Zhang et al., 2018), comparing different injections for a specific clinical question in one cancer type, or animal studies (Zhu et al., 2019) or *in vitro* study (Zheng et al., 2020).

### Limitations

Several limitations existed in the study. Firstly, a complete assessment of study quality for all included RCTs and SRs was not manageable, therefore only key components were used as a compromise. Secondly, we did not contact the authors of the primary trials to understand the most unclear risk of bias in the implementation of the trials. Some trials with low risk of bias may not have been included due to incomplete reporting, so the evidence mapping may not be sufficiently comprehensive to reflect the whole quality of evidence in this field. Thirdly, the study selection and data extraction were not done independently by two persons, which may lead to a possible selection bias.

### Implications for Future Research

Several implications could be raised for future research in Chinese herbal injections for cancer care. Firstly, the evidence mapping presented the evidence gap on overall and progress-free survival. Though tumor response was also reported in some trials, it was only an alternative outcome and could not fully represent clinical benefit. Large-scale observational studies or RCTs with a well-designed follow-up period are warranted (Xu et al., 2017). Secondly, outcomes on quality of life showed the most consistent beneficial effects among different cancer types. However, most of them were evaluated by KPS scores, which is not a comprehensive scale for quality of life as it reflected the functional status (Terret et al., 2011). Research focusing on this area with latest scales or patient-reported outcomes, for example, the Patient-Reported Outcomes version of the Common Terminology Criteria for Adverse Events (PRO-CTCAE), would help doctors and patients know more advantages and disadvantages of the injections on quality of life (Kluetz et al., 2016). Thirdly, the trials seldom reported the syndrome differentiation. Though there is still no consensus on whether it is needed for all herbal injections, eight of ten included injections used expressions in traditional Chinese medicine theory for the description of indications, which suggested most herbal injections in cancer care need syndrome differentiation. The low percentage showed a severe lack of reporting and was a limitation of the study design. Fourthly, in a majority of the included trials, herbal injections were used combined with chemotherapy drugs. Though some of the trials discussed the effect of “increased efficiency and reduced toxicity”, it was not clear enough. The knowledge gap of the herb-drug interaction needs more attention and further clinical studies of pharmacokinetics and pharmacodynamics in the combined use of herbal injections and chemotherapy are extremely warranted.(Yeung et al., 2018).

### Implications for Clinical Practice and Policy Making

The evidence mapping suggested the possible clinical status of the included injections. Compound Kushen, Aidi, Kanglaite, and Cinobufacins injections have anti-tumor effects and appear to have beneficial survival outcomes. Shenqi Fuzheng and *Astragalus* polysaccharide injections have adjuvant treatment effects. Although there was a certain benefit to the objective response rate of tumors, Kangai, Xiaoaiping, and *Brucea javanica* oil emulsion injections need further trials to optimize the characteristics to clarify the clinical indications due to the limited survival outcome reported. As there is an obvious lack of evidence, it is difficult to support the clinical indications for *Polyporus* polysaccharide injections.

For clinical practice, this evidence mapping presents a precise and comprehensive summary of the clinical application of Chinese herbal injections: Aidi for lung and liver cancer; Cinobufacin for liver cancer; Compound Kushen and *Brucea javanica* oil emulsion for gastrointestinal cancer, especially for pancreatic cancer; Compound Kushen for radiation pneumonitis; Xiaoaiping for quality of life in lung cancer; the adjuvant effect of Shenqi Fuzheng for myelosuppression; Kangai for liver function; and Kanglaite to support immune function. These results were drawn from RCTs with consistent findings, showing very promising effects. However, in clinical practice, the current health insurance regulations and drug indications are currently too broad to support a clinician’s choice of a specific treatment. Given the promising findings in this evidence mapping, the use of these injections could be wider if supported by further large well-designed trials or real world studies.

The research on time and duration of herbal injections’ administration still needs more exploration. RCTs on the early use of herbal injections before standard treatment began were rare, at less than 10%. At the same time, the results showed that herbal injections had advantages on the improvement of myelosuppression and immune function, which were critical indicators of whether to continue treatment before and during chemotherapy. Due to drug indications and medical insurance regulations, it is not possible to use herbal injections in advance to achieve and maintain the above functions so as to help ensure the successful completion of chemotherapy.

As Table 2 presented, over 40% of the included herbal injections were used for the same period as standard treatment, such as intravenous administration for 21 days. Some of the drug indications were also used in consecutive 21–30 days of treatment. As far as we know, it was different from the clinical reality as the patients asked to be discharged from hospital when they finished the current cycle treatment and were unable to receive the herbal injections in outpatient departments or in primary care, as eight of ten injections within the health insurance program were limited use in secondary or tertiary hospitals. As a result, the gap between drug indication, research evidence, and clinical practice needs more consideration and clarification.

For policy making, some of the outcomes and effects of injections in the anti-tumor category and adjuvant cancer treatment category were very similar based on the included evidence, but there would be differences in the medical insurance payment according to the category. In addition, health economics research based on high-quality evidence is urgently needed to compare the clinical effects, side effects, and comprehensive drug costs of different injections, so as to provide more objective evaluation for decision-making.

## Conclusion

Since the 21st century, high-level evidence including RCTs, systematic reviews, and meta-analyses of Chinese medicine injection in cancer care has gradually increased. The current evidence mapping provided a visual overview of the beneficial effects for herbal injections in cancer care. The clinical benefits and evidence gaps offered useful information for different stakeholders and for informing policy and clinical decision makers.

## Data Availability

All data generated and analyzed during this study are included in this article/Supplementary Material. The included studies were published on the open access website and databases.
