# Supplementary material for: Clinical Application of Chinese Herbal Injection for Cancer Care: Evidence-Mapping of the Systematic Reviews, Meta-analyses, and Randomized Controlled Trials
Source: Front Pharmacol. 2021 May 7;12:666368. doi: 10.3389/fphar.2021.666368 (PMC8138123; doi:10.3389/fphar.2021.666368)
Supplement: Supplementary file 2 [file Table1.doc]

**Supplementary Table 1 Detailed information on the ten herbal injections**

| **Chinese herbal injection** | **Source** | **Raw material** | **Phytochemical compositions** | **Therapeutic claims in TCM** | **Indications** | **Quality control reported? (Y/N)** | **Chemical analysis reported? (Y/N)** |
| --- | --- | --- | --- | --- | --- | --- | --- |
| Compound kushen injection | Shanxi Zhendong Pharmaceutical Co., Ltd | Sophora flavescens Aiton (Kushen), Smilax glabra Roxb. (Baituling) | Oxidative sophorine, oxymatrine and matrine, etc | Clearing heat and draining dampness, cooling the blood and resolving toxin, dissipating binds and relieving pain;  Indications: Cancer pain and bleeding. | Pain caused by cancer, bleeding | Y - National Food and Drug Administration National Drug Standards | **N** |
| Shenqi fuzheng injection | LIVZON Pharmaceutical Group Co., Ltd. | Extracts from Codonopsis pilosula (Franch.) Nannf. (Dangshen)*,* Astragalus: Astragalus membranaceus (Fisch.) Bunge. (Huangqi) | Astragaloside I, Astragaloside II, Astragaloside III, Astragaloside IV, Lobetyolin, Formononetin, etc. | Boosting qi and reinforcing the healthy qi;  Indications: Fatigue, asthenic breathing, laziness to speak, spontaneous sweating and dizziness caused by lung-spleen-qi deficiency; adjuvant treatment for lung cancer and gastric cancer with the above symptoms | Fatigue, lack of strength, vertigo caused by asthenia of pulmonosplenic *qi*; auxiliary treatment of the above symptoms for lung cancer and gastric cancer, etc. | Y - National Food and Drug Administration National Drug Standards | **N** |
| Aidi  Injection | Guizhou Yibai Pharmaceutical Co. Ltd | Astragalus: Astragalus membranaceus (Fisch.) Bunge. (Huangqi), Eleutherococcus senticosus: Acanthopanax senticosus (Rupr. Maxim.) Harms (Ciwujia), Ginseng: Panax ginseng C. A. Mey. (Renshen),  Mylabris phalerata (Pallas)  (Banmao) | Astragaloside II  Astragaloside IV  Cantharidin  Ginsenoside Rb1  Ginsenoside Rg1  Ginsenoside Rg3  Ginsenoside Rh2  Ginsenoside Rc  Ginsenoside Rd  Ginsenoside Re  Isofraxidin  Syringin, etc | Clearing heat and removing toxin, eliminating blood stasis and dissipating binds;  Indications: Primary liver cancer, lung cancer, rectal cancer, malignant lymphoma, gynecological malignancy, etc. | Primary liver cancer, lung cancer, rectal cancer, malignant lymphoma, gynecological malignancy, etc | Y - National Food and Drug Administration National Drug Standards | **N** |
| Kangai injection | Changbai Mountain Pharmaceutical Co. Ltd | Astragalus: Astragalus membranaceus (Fisch.) Bunge. (Huangqi), Ginseng: Panax ginseng C. A. Mey. (Renshen), Ammothamnine | Astragaloside, ginsenoside Rg1,  ginsenoside Re,  ginsenoside Rf,  ginsenoside Rb1,  Ammothamnine, etc | Boosting qi and reinforcing the healthy qi, strengthening the body's immune function;  Indications: Primary liver cancer, lung cancer, rectal cancer, malignant lymphoma, gynecological malignant tumors; Leukopenia and hypoxia caused by various reasons; Chronic hepatitis B. | Primary liver cancer, lung cancer, rectal cancer, malignant lymphoma, gynecological malignancy, leukocyte hypoplasia and reduction caused by various reasons, chronic hepatitis B, etc | Y - National Food and Drug Administration National Drug Standards | **N** |
| Kanglaite injection | Zhejiang Kanglei Pharmaceutical Co. Ltd | Oil from Coix lacryma-jobi L. for injection (Oil from Yiyiren) | Coix seed ester,  Coixol (C_8_H_7_O_3_N), palmitic acid,  stearic acid,  myristic acid, etc. | Boosting qi and nourishing yin, eliminating mass and dissipating binds; Indications: Primary non-small cell lung cancer and primary liver cancer with qi-yin deficiency pattern or spleen deficiency and dampness encumbrance pattern; It has a certain synergistic effect with radiotherapy and chemotherapy; It has certain anti-cachexia and analgesic effects for patients the medium and advanced tumors. | Unsuitable for operation of Qi and Yin deficiency, spleen deficiency wet trapped type of primary non-small cell lung cancer and primary liver cancer. It has certain synergistic effect in combination with radiotherapy and chemotherapy. It has a certain anti-cachexia and analgesic effect on middle and advanced tumor patients. | Y - National Food and Drug Administration National Drug Standards | **N** |
| Tongguanteng(Xiaoaiping) injection | Nanjing Shenghe Pharmaceutical Co.,Ltd. Tonghua Golden-Horse Pharmaceutical Industey Co.,Ltd | Marsdenia tenacissima (Roxb.) Moon (Tongguanteng) | 17β-tenacigenin B,  Tenacigenin B(TB),  Tenacigenin A(TA),  TenacigenosideA(TSA),  Marsdenoside I(MSI),  Tenacissoside F(TSF) | Clearing heat and removing toxin, resolving phlegm and softening hardness;  Indications: It is used for esophageal cancer, gastric cancer, lung cancer, liver cancer, and can be used as adjuvant treatment for radiotherapy and chemotherapy. | Esophageal cancer, gastric cancer, lung cancer, liver cancer, can be combined with radiotherapy, chemotherapy as adjuvant therapy. | Y - National Food and Drug Administration National Drug Standards | **N** |
| Cinobufacin injection | Anhui China Resources Jinchan Pharmaceutical Co., Ltd | Cinobufacin | Cinobufacin | Removing toxin, dispersing swelling and relieving pain;  Indications: Middle and advanced tumors, chronic hepatitis B and other diseases. | Advanced tumor, chronic hepatitis B. | Y - National Food and Drug Administration National Drug Standards | **N** |
| *Brucea javanica* oil emulsion injection | Jiangsu Jiuxu Pharmaceutical Co., Ltd., Shenyang Pharmaceutical Da Lei Yunshang Pharmaceutical Co., Ltd | Oil emulsion from Brucea javanica (L.) Merr. (Yadanzi) | Palmitic acid, stearic acid, oleic acid and linoleic acid, etc | Anti-cancer drug  Indications: Lung cancer, lung cancer brain metastases and digestive tract tumors. | Lung cancer, lung cancer brain metastases, and digestive tract tumors. | Y - National Food and Drug Administration National Drug Standards | **N** |
| *Polyporus* polysaccharide injection | Chia Tai Tianqing Pharmaceutical Group Co. Ltd | Polyporus polysaccharide | polyporus polysaccharide | It can regulate the immune function of the body and has a certain effect on chronic hepatitis and tumors. Combined with anti-tumor chemotherapy drugs, it can enhance the efficacy and reduce toxic side effects | Regulating body immune function, treating chronic hepatitis, tumor. Combined with antitumor chemotherapeutic drugs, it can enhance the curative effect and reduce the toxic and side effects. | Y - National Food and Drug Administration National Drug Standards | **N** |
| *Astragalus* Polysaccharide for injection | Tianjin Sainuo Pharmaceutical Co., Ltd | Astragalus Polysaccharide | Astragalus Polysaccharide | Supplement qi deficiency. | It is used for cancer patients with reduced white blood cells, reduced quality of life and weakened immune function after chemotherapy. | Y - National Food and Drug Administration National Drug Standards | **N** |

**References:**

Wu D, Liang J, Lian JW, Zhang L, Chen XH, Bi KS. (2006). Simultaneous RP-HPLC determination of oxysophocarpine oxymatrine and matrine in compound kushen injection. Journal of Shenyang Pharmaceutical University.23(4), 220-223

Shu, J, and Yang SB. (2016). Simultaneous determination of six components in shenqi fuzheng injection by HPLC. China Pharm. 27(30), 4295-4297.

Zhang MM, Liu YL, Chen C, Li XR, Xu QM, Yang SH.(2012). Studies on chemical constituents from Aidi Injection. Chin Tradit Herb Drugs 43(8) (2012) 1462-1470.

Zeng YB, Liu XL, Li CJ, Zhou X, Zhang XM, Yang Y, et al. (2016). Chemical constituents from Mylabris phalerata and their cytotoxic activity in vitro, J Chin Mater Med 41(5), 859-863.

Yang Z, Zhang Y, Zhu YJ, Sun ZP, Li XR. (2011). UPLC-MS/MS Determination of five main components in Kangai injection. Chin Pharm J. 46(4), 297-299.

Wen RX. (2008). Progress in study of Chemical constituents and anti-tumor activities of semen coicis. Journal of Liaoning University of TCM. 10(3),135-138

Gong XB, Zeng QH, Zhang F, Gao SH, Lu WQ, Chen WS. (2016). Fingerprint Analysis of XiaoaipingInjection and Simultaneous Determination of Its Seven Active Steroidal Components in XiaoaipingInjection by HPLC-ELSD. Chinese Journal of Experimental Traditional Medical Formulae.22(15), 68-72

Liu D, Han F, Li S, Bi KS, Chen XH.(2012). Capillary GC Simultaneous Determination of 4 Fatty Acids in Brucea Javanica Oil Latex Injection. China Pharmacy.23(4), 364-366
